# Sequential endoscopic and robot-assisted surgical solutions for a rare fungal spondylodiscitis, secondary lumbar spinal stenosis, and subsequent discal pseudocyst causing acute cauda equina syndrome: a case report

**DOI:** 10.1186/s12893-022-01493-3

**Published:** 2022-01-29

**Authors:** Chao Wang, Lu Zhang, Hao Zhang, Derong Xu, Xuexiao Ma

**Affiliations:** Department of Spine Surgery, The Affiliated Hospital of Qingdao University, 59 Haier Road, Qingdao, 266000 Shandong China

**Keywords:** Endoscopic surgery, Robot-assisted surgery, Fungal spondylodiscitis, Discal pseudocyst, Cortical bone trajectory, Pedicle screw

## Abstract

**Background:**

Fungal spondylodiscitis is a rare infectious disease. The secondary lumbar spinal stenosis and postoperative discal pseudocyst were even rarer. The surgical interventions were disputed, yet endoscopic and robot-assisted techniques may be helpful under different circumstances.

**Case presentation:**

A 62-year-old female was diagnosed as infectious spondylodiscitis at the L4/5 level and a posterolateral endoscopic debridement was performed after invalid conservative therapy. Causative organism culture revealed a rare fungus, *Candida* *tropicalis*. A secondary spinal stenosis with refractory radiculopathy occurred almost 3 years after the first surgery and a successful endoscopic surgery was implemented aiming to decompress the nerve in a minimally invasive way. However, 2 months later, the patient manifested severe acute cauda equina syndrome and radiological examinations suggested a rare postoperative discal pseudocyst. A laminectomy followed by a pseudocystectomy was applied to achieve thorough decompression. An innovative double trajectory system (simultaneous traditional pedicle screw and cortical bone trajectory screw) accompanied by posterolateral fusion was designed and executed by the professional robot-assisted system.

**Conclusion:**

Endoscopic and robot-assisted techniques may provide alternative solutions for fungal spondylodiscitis and accompanied sequelae.

## Background

Fungal spondylodiscitis is a rare infectious disease which is often difficult to tackle for spine surgeons. The patient usually presents immunosuppressive or immunocompromised state associated with immunodeficiency disease, corticosteroid usage, chemotherapy, diabetes mellitus, or malnutrition [1, 2]. Thus, traditional open surgery is challenging for its high risk of uncontrollable infection. Endoscopic technique was firstly introduced into spine surgery at 1980s for disc herniation. Over the last decade, the technique was explored and implemented successfully in the field of other spinal diseases such as canal stenosis and spinal infection. Similarly, endoscopic surgery provides a novel scope for fungal spondylodiscitis and the accompanied sequelae.

Herein, we presented a sophisticated and instructive case who was initially diagnosed as fungal spondylodiscitis after endoscopic debridement and organism culture. The secondary lumbar spinal stenosis was resolved by a similar endoscopic procedure considering the willing of the patient and the well stability of the local vertebrae. After identifying a rare postoperative discal pseudocyst (PDP) at the same level causing acute cauda equine syndrome, we performed a pseudocystectomy and posterolateral fusion together with instrumentation. The screw fixation was aided by a robot-assisted system for the design and execution of a unique double trajectory system for the defect bone trajectory of the vertebrae. To our best knowledge, this is the first case report in literature in the following aspects: (1) sequential endoscopic surgeries for both fungal spondylodiscitis and secondary spinal stenosis; (2) robot-assisted double trajectory system for screw augmentation in the defect trajectory case.

## Case presentation

A 62-year-old female complained of low back pain for 1 month and referred to our institute in April 2016. Previously, the patient was diagnosed as acute leukemia (M2 type) due to fatigue accompanied by remarkably elevated white blood cells in August 2015. From August 2015 to December 2016, she received nine courses of chemotherapy. During this period, she was diagnosed as oral candidiasis because of intermittent fever, oral leukoplakia and ulceration. She received fluconazole and voriconazole treatment successively. However, the anti-fungal courses were interrupted unintendedly because of the digestive side effects and liver function damage.

**Fig. 1:**
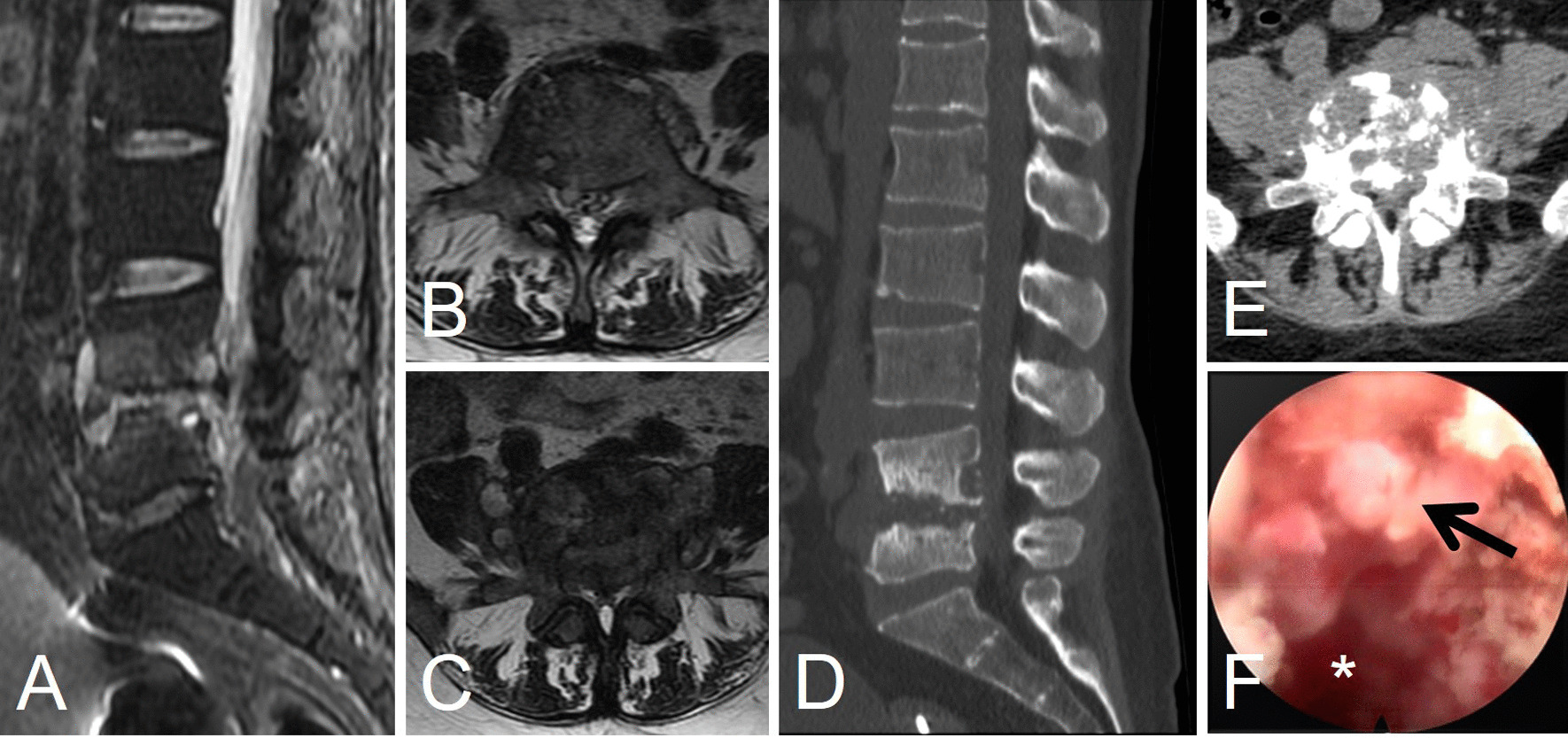
Images of the lesion before or at the first endoscopic surgery (debridement). The MR images of initial administration (**A**, **B**), the MR (**C**) and CT images (**D**, **E**) at 5 months after empirical treatment, and endoscopic view of the discal lesion at surgery (**F**) were presented. The star indicates the interbody space and the arrow indicates inflammatory tissue

On physical examination, the patient had a limited range of motion at the lumbar spine and tenderness at L4–5 level. No positive sign was detected for both lower extremities. Serum inflammatory indicators, such as erythrocyte sedimentation rate (ESR), C-reactive protein (CRP), and procalcitonin (PCT) showed significant elevation. Immediate magnetic resonance imaging (MRI) showed collapsed L4–5 disc space and hyperintense on T2-weighed imaging (T2WI) at both intervertebral and paravertebral space, which was speculated to be a suppurative spondylodiscitis (Fig. 1A, B). Empirical anti-microbial treatment was implemented for 5 months, but the back pain got worse and subsequent MRI verified that the lesion further expanded to paravertebral psoas space (Fig. 1C). Computed tomography (CT) depicted more detailed information upon the end plate destruction and vertebral erosion (Fig. 1D, E).

Considering the immunosuppressive condition of the patient, an open surgery was abandoned for its high risk of uncontrollable infection. Finally, a posterolateral endoscopic discectomy was adopted to debride the necrotic lesion and to acquire enough tissue for organism culture (Fig. 1F). The culture result revealed a rare fungi species, *Candida tropicalis*. Antifungal drug susceptibility test indicated that amphotericin B was the most suitable drug (Table 1). The patient experienced immediate pain relief after surgery. Systematic anti-fungal drugs together with supportive therapy for more than 1 year showed further clinical improvement and laboratory effectiveness.

**Table 1:** Results of organism culture and drug susceptibility test

Organism culture	*Candida tropicalis*	
Drugs	Susceptibility	MIC
5-flucytosine	Sensitive	≤ 4
Amphotericin B	Sensitive	1
Fluconazole	Resistant	4
Itraconazole	Resistant	4
Voriconazole	Resistant	> 8

*MIC* Minimum inhibitory concentration

**Fig. 2:**
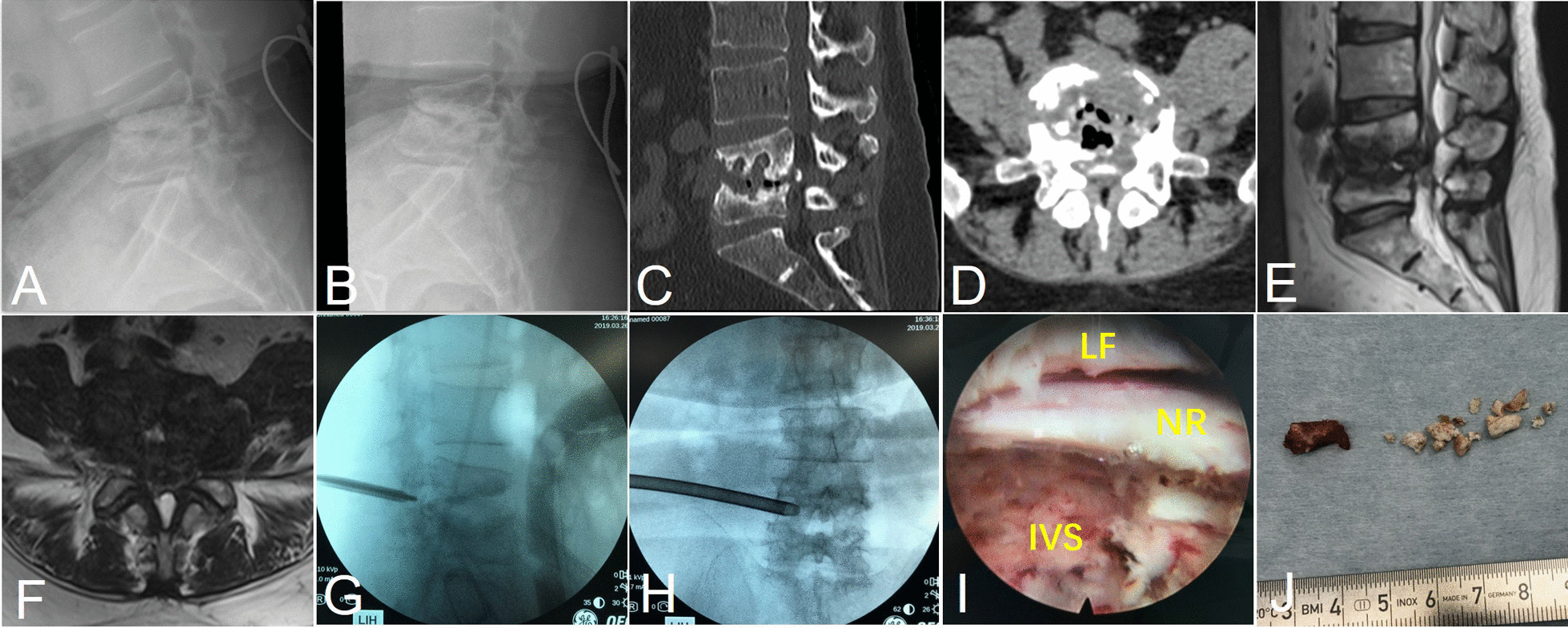
Images of the lesion before or at the second endoscopic surgery (foraminoplasty and discectomy). The dynamic roentgenography (**A**, **B**), CT (**C**, **D**), and MR T2WI (**E**, **F**) images of the second administration, intraoperative localization (**G**, **H**), and endoscopic view (**I**) after foraminoplasty and discectomy (**J**) were presented. *LF* ligamentum flavum, *NR* nerve root, *IVS* intervertebral space

**Fig. 3:**
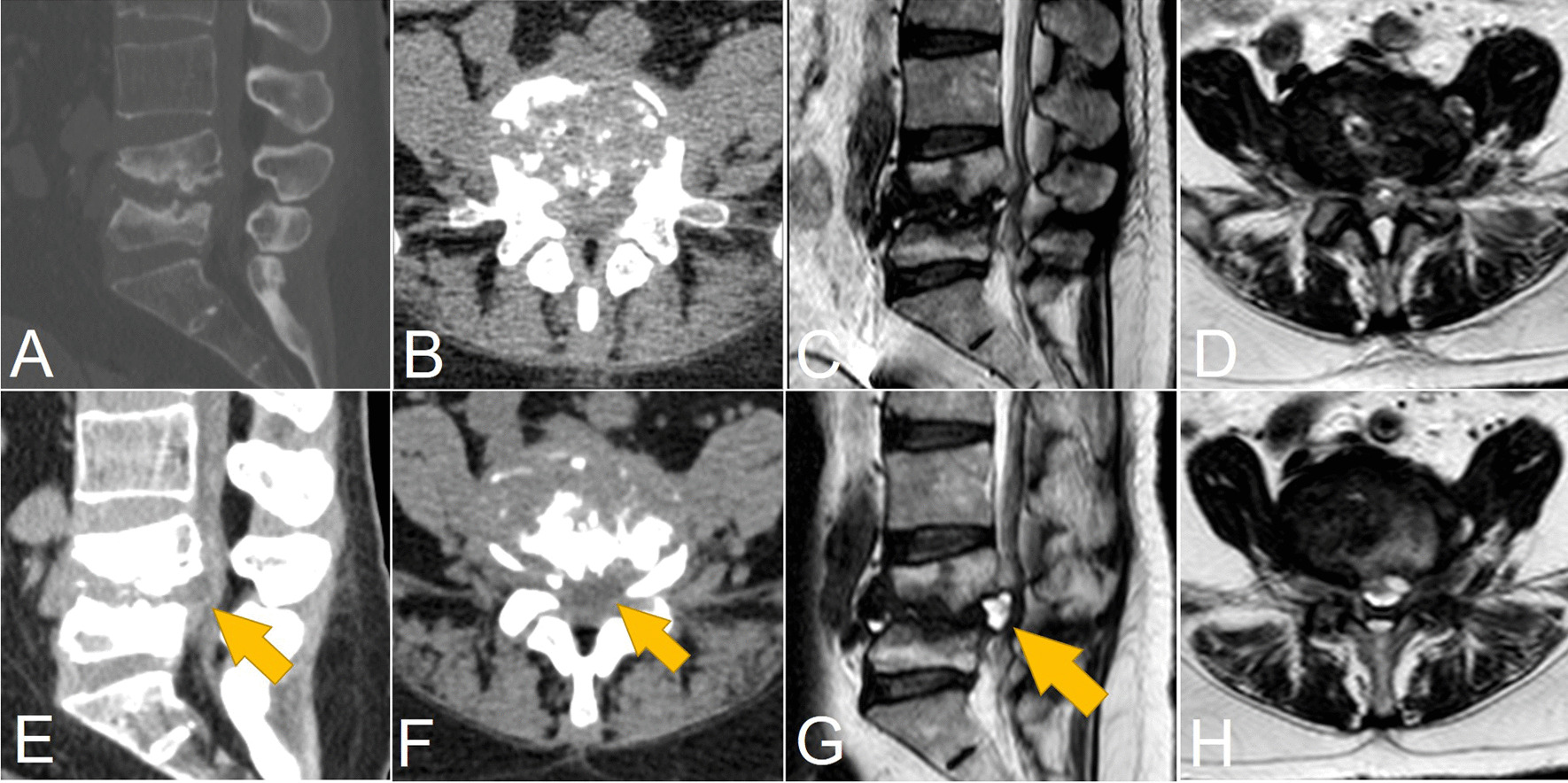
Comparison of CT/MRI images after the second and before the third operation. CT (**A**, **B**) and MRI (**C**, **D**) images after the second operation. CT (**E**, **F**) and MRI (**G**, **H**) images before the third operation. Arrow indicated the manifestation of the pseudocyst on CT (**E**, **F**) and MRI (**G**)

However, the patient reported aggravated intermittent claudication and numbness at the left L5 sensory area in March 2019. The white blood cell counting, CRP, ESR, and PCT were all normal at this time. Dynamic roentgenography confirmed the local kyphosis but no evidence of lumbar instability (Fig. 2A, B). Compared to the previous imaging, CT scan and MRI showed further collapse of the disc space and bony destruction (Fig. 2C–F). Sclerosis at the edges was prominent and no hyperintense T2WI at or around L4–5 level was observed, which suggested the primary elimination of the fungal infection. Therefore, secondary spinal stenosis was considered. Since the patient was in fear of open surgery and firmly requested minimally invasive surgery, we conducted a transforaminal approach endoscopic foraminoplasty and discectomy (Fig. 2G–J). The nerve root and dual sac decompression was confirmed by intraoperative visualization and postoperative CT scan and MRI (Fig. 3A–D). The patient was satisfied with the procedure and discharged uneventfully.

Unfortunately, the patient returned back again 2 months after her second operation because she experienced rapidly progressed low back and leg pain as well as gut and urine dysfunction. She was immediately arranged for emergency CT scan and MRI. Surprisingly, both CT and MRI presented a cyst-like lesion in the lumbar spinal canal at the L4–5 disc level (Fig. 3E–H). The cyst had close connection with the disc, much alike the discal pseudocyst. Since the patient manifested symptoms of cauda equina syndrome, a thorough decompression surgery, i.e., laminectomy and pseudocystectomy together with instrumentation and fusion, was planned after discussions among surgeons and a sufficient informed consent with the patient and her family.

**Fig. 4:**
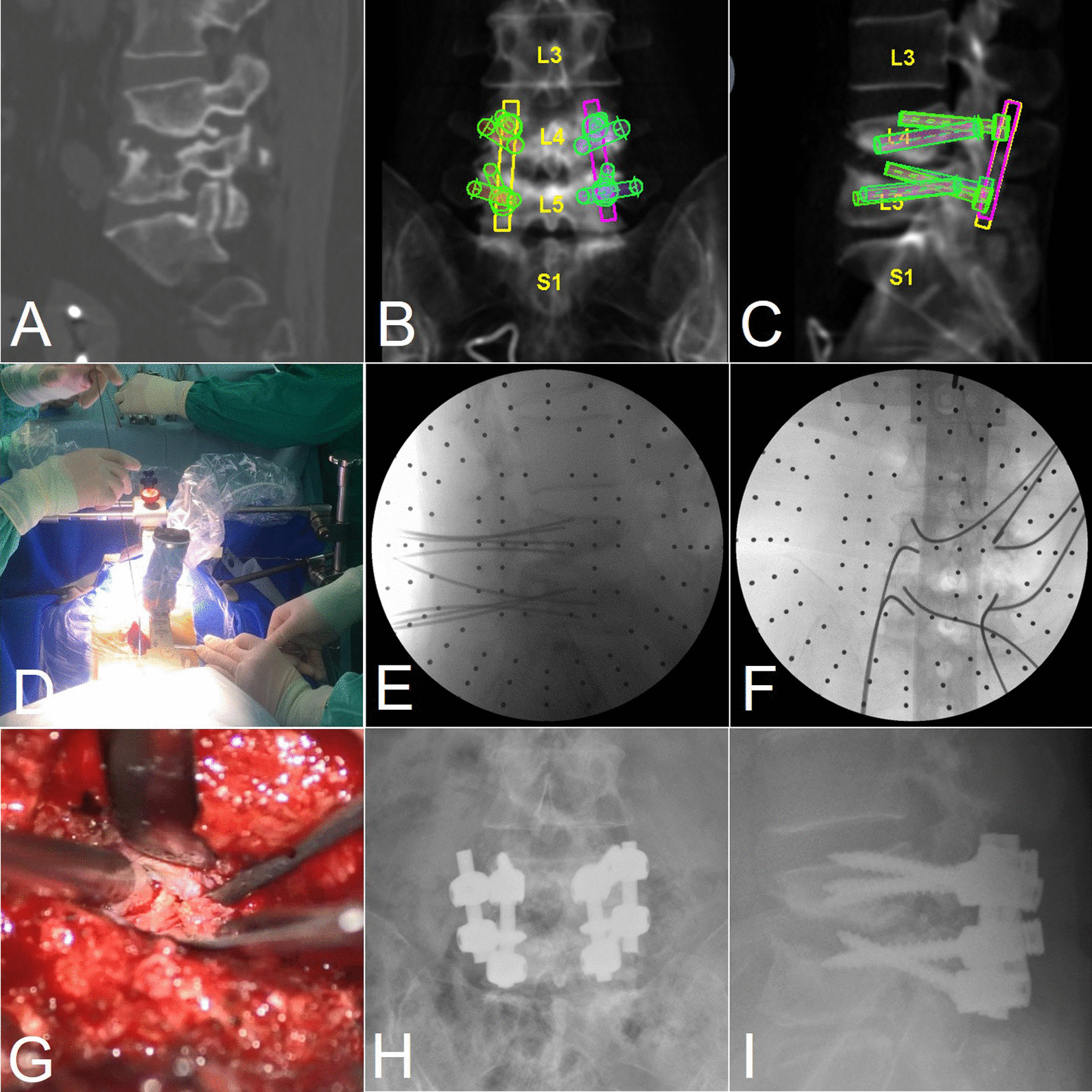
Images of preoperative CT scan (**A**) and screw simulation (**B**, **C**), intraoperative K-wire insertion under guidance of robotic system (**D**–**F**) and microscopic view of the pseudocyst (**G**), and postoperative roentgenography showing position of the screws (**H**, **I**)

For this case, there were two technical challenges encountered by spinal surgeons. The bony capacity for screws, either the traditional pedicle (TP) screw or the cortical bone trajectory (CBT) screw, reduced because the erosion by the previous infection, especially at the L5 vertebra (Fig. 4A). Therefore, strength of either instrumentation system was predicted to be biomechanically insufficient. Hence, we utilized the Renaissance robotic system (Mazor Robotics Ltd.) to design a unique “double trajectory system” which contained both TP and CBT screw systems at the same level (Fig. 4B, C). Benefit from the cautious preoperative planning and precise intraoperative execution, the K-wire and cannulated screws were eventually inserted successively (Fig. 4D–F). Another challenge was the pattern for spinal fusion. As obviously shown in preoperative CT scan (Fig. 3E), the osseous interface between L4 and L5 vertebral bodies became sclerotic and rugged, which made the interbody fusion difficult and indefinite. Consequently, we decided to implement a more feasible posterolateral fusion instead.

After the laminectomy and elaborative dissection between the dural sac and the pseudocyst, a piecemeal pseudocystectomy was completed (Fig. 4G). The content of the cyst was serous and the pathology for the cyst wall showed hyperplasia of fibrous tissue without typical presentation of lining cells.

**Fig. 5:**
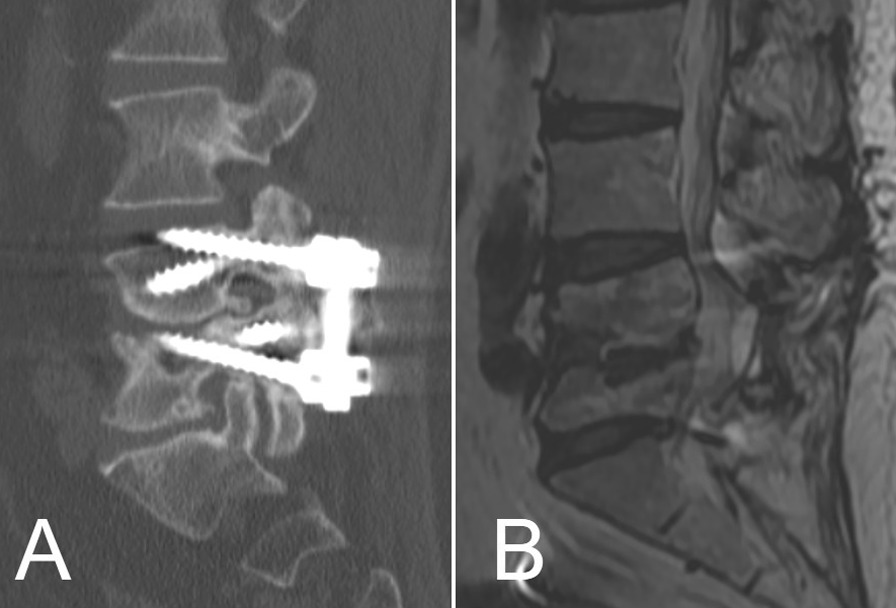
The CT (**A**) and MRI (**B**) at 2.5-year follow-up. CT showed ultimate bony fusion of the L4–5 segments and penetration of a CBT screw into the L3–4 interbody space. MRI showed no recurrence but adjacent segmental degeneration and asymptomatic canal stenosis

The patient felt instant relief on her back and leg pain after surgery. Postoperative roentgenography confirmed the positions of implanted screws (Fig. 4H, I). She felt recovery of her gastrointestinal and urine function except mild numbness at perineum after 1 month. The patient was followed up for 2.5 years and no evidence of recurrence was observed. The bony fusion was observed at the sagittal CT scan (Fig. 5A). The adjacent L3–4 degeneration and canal stenosis was also obvious but it was asymptomatic (Fig. 5B). The perioperative information of the three surgeries was listed as Table 2.

**Table 2:** Perioperative information of the three surgeries for the patient

Surgery no	1	2	3
Preoperative			
VAS	7	7	9
ODI	74	78	88
Primary diagnosis	Fungal spondylodiscitis	Lumbar spinal stenosis	Postoperative discal pseudocyst
Surgery	Endoscopic debridement	Endoscopic foraminoplasty and discectomy	Laminectomy, pseudocystectomy, robot-assisted instrumentation, and PLF
Intraoperative			
Anesthesia	Local	Local	General
EBL (mL)	10	5	200
Duration (min)	105	125	255
Postoperative			
VAS	3	2	2

*VAS* Visual Analogue Scale, *ODI* Oswestry Disability Index, *EBL* estimated blood loss, *PLF* posterolateral fusion

## Discussion and conclusion

Fungal spondylodiscitis is a rare spinal infectious disease, of which Aspergillus and Candida are the most commonly identified species [2, 3]. Most of the fungal species are normal commensals colonized at surface of the skin or mucosa but may become detrimental when they are translocated by the venous system in susceptible conditions such as immunosuppression, immunodeficiency, and antibiotic abuse. Although sharing similar management principle and algorithm with the more common pyogenic, tuberculous, and brucellar infection, it has characterized imaging features [1]. Nonetheless, as lacking specific diagnostic methods, it is usually a necessity to obtain the pathogenic evidence for the targeted antibiotic therapy. Whether by CT-guided biopsy or surgery, microbial culture of the infectious tissue and subsequent drug sensitivity test are the pivotal routes to the ultimate recovery. After reviewing 130 articles published between 1948 and 2010, Ganesh et al. [2] concluded that patients treated by combined surgery and antifungal therapy had better clinical outcomes than patients underwent medical therapy alone.

Recently, endoscopic debridement and irrigation has been reported as an alternative solution for spinal infection, especially in patients with severe comorbid conditions [4–7]. After retrieving the PubMed database, we found only three articles (including six cases) depicting endoscopic technique in fungal spondylodiscitis [8–10]. Even though the causative organisms varied from the common Candida to rarely mentioned Scedosporium, the authors shared similar posterolateral approach and principles for debridement, irrigation, and drainage as Ito et al. described in their paper [5]. All the cases recovered successfully at the final follow-up, which suggests that the procedure is effective and minimally invasive in treatment of fungal spondylodiscitis. For this patient, the abscess in the psoas was not deliberately eliminated considering the difficulty of the procedure and the self-limited course after utilization of the susceptible antibiotic. Postoperative irrigation and drainage was not implied since the narrowing of the interbody space makes it hard for a proper tube position and an efficient drainage system. Similar to the patient whom Fan and colleagues [8] described in their paper, the second endoscopic surgery for our patient was carried out because of the secondary lumbar canal stenosis to the postinfectious histological and biomechanical changes. Therefore, the key point for the second endoscopic surgery should be canal and lateral recess decompression, rather than infection debridement and organism culture which were indeed the targets of our first endoscopic surgery.

Postoperative discal pseudocyst, or named “annular pseudocyst” by Young et al. [11] in their case report for the first description, is a rare cause for relapsed or even worse symptoms after various discectomies, such as microdiscectomy, microendoscopic discectomy, and percutaneous endoscopic discectomy. MRI usually manifested T2WI hyperintense of the collections and communicated with the disc annulus [11]. Kang et al. [12] investigated MRIs of 1503 male soldiers receiving endoscopic lumbar discectomy and found that PDP was diagnosed in approximately 1.0% of the initial cases and the mean interval from surgery to PDP detection was 53.7 days. Though the mechanism of the PDP formation was not clear, nonsurgical treatment was the first choice for most symptomatic cases. Chung and colleagues [13] retrospectively reported 12 PDP patients, of which half patients underwent conservative therapy and spontaneous regression of the lesion was observed. Fu et al. [14] achieved similar result for their patient by successful conservation. Interventional therapy is effective and can be considered for patients with invalid conservative treatment. One of the two PDP cases after microdiscectomy reported by Young et al. [11] experienced remarkable relief after the aspiration and local injection of steroid. Cystectomy is indicated for patients with neurological deficits or when conservative treatment is ineffective [13, 15].

Our patient presented a PDP with acute cauda equina syndrome, which to our best knowledge is not previously reported in literature. Under this circumstance, a thorough ventral and dorsal decompression rather than a simple cystectomy, would be the first and foremost consideration. Therefore, an additional instrumentation and fusion would be necessary. One reasonable strategy for the defective or osteoporotic bony trajectory is to increase the number of segment for fixation. Nevertheless, this solution sacrifices the movements of the adjacent segments and brings side effects of long segment fixation such as proximal junctional kyphosis/failure, pseudoarthrosis, and extensive soft tissue injury. Therefore, we designed a double trajectory system which could accommodate both kinds of screws in one pedicle. Previously, we have employed the robot-assisted system at multiple scenarios [16]. Relying on unique preoperative planning and precise intraoperative implantation, the robot-assisted surgery exhibits outstanding advantages in simultaneous double trajectories with no screw conflict. In 2013, the double trajectory technique was firstly proposed by Ueno et al. in an osteoporotic patient with degenerative lumbar scoliosis by a “freehand” manner [17]. However, since the entry point of the CBT screw was elevated and the cranial angle was decreased compared to the classical trajectory, the biomechanical function of the CBT screw engaging the dense cortical bone was prominently impaired. Even though Matsukawa and colleagues [18] demonstrated the overwhelming biomechanical advantages of the double trajectory technique compared to either CBT or TP technique by finite element analysis, the technique was seldom referred to afterwards probably because of the concerns on potential pedicle burst and screw conflict. Our experience indicates that this technique combined with robot technique may provide a novel solution for defective or osteoporotic bone trajectory.

There are several limitations in the treatment of this patient. Though the CT scan at follow-up showed the solid posterolateral fusion, it simultaneously revealed the penetration of the upper CBT screws to the endplate of L4 (Fig. 5A). The MRI confirmed an asymptomatic adjacent segmental stenosis, which might be related to the disc space violation.

In conclusion, we reported a rare fungal spondylodiscitis and secondary lumbar spinal stenosis treated sequentially by endoscopic surgeries. A novel robot-assisted double screw technique was used for the instrumentation of the subsequent discal pseudocyst causing acute cauda equina syndrome. Endoscopic and robot-assisted techniques provide alternative solutions for fungal spondylodiscitis and accompanied sequelae.

## Data Availability

All available data was included in this published case report.

## Abbreviations

PDP: Postoperative discal pseudocyst
ESR: Erythrocyte sedimentation rate
CRP: C-reactive protein
PCT: Procalcitonin
MRI: Magnetic resonance imaging
T2WI: T2-weighed imaging
CT: Computed tomography
TP: Traditional pedicle
CBT: Cortical bone trajectory
VAS: Visual Analogue Scale
ODI: Oswestry Disability Index
EBL: Estimated blood loss
PLF: Posterolateral fusion
MIC: Minimum inhibitory concentration
LF: Ligamentum flavum
NR: Nerve root
IVS: Intervertebral space
